# The (cost-)effectiveness and cost-utility of a novel integrative care initiative for patients with chronic musculoskeletal pain: the pragmatic trial protocol of Network Pain Rehabilitation Limburg

**DOI:** 10.1186/s12955-020-01569-9

**Published:** 2020-10-01

**Authors:** Cynthia Lamper, Ivan P. J. Huijnen, Mariëlle E. J. B. Goossens, Bjorn Winkens, Dirk Ruwaard, Jeanine A. M. C. F. Verbunt, Mariëlle E. Kroese

**Affiliations:** 1 Department of Rehabilitation Medicine, School for Public Health and Primary Care (CAPHRI), Faculty of Health, Medicine and Life Sciences, Maastricht University, Universiteitssingel 40, 6229 ER Maastricht, P.O. Box 616, 6200 MD Maastricht, The Netherlands; 2 Centre of Expertise in Rehabilitation and Audiology, Adelante, Hoensbroek, The Netherlands; 3 Department of Methodology and Statistics, Care and Public Health Research Institute (CAPHRI), Maastricht University, Maastricht, The Netherlands; 4 Department of Health Services Research, School for Public Health and Primary Care (CAPHRI), Faculty of Health, Medicine and Life Sciences, Maastricht University, Maastricht, The Netherlands

**Keywords:** Effectiveness, Cost-effectiveness, Cost-utility, Pragmatic trial, Rehabilitation, Chronic musculoskeletal pain, Organization of care, Primary care, Network

## Abstract

**Background:**

Rehabilitation care for patients with chronic musculoskeletal pain (CMP) is not optimally organized. The Network Pain Rehabilitation Limburg 2.0 (NPRL2.0) provides integrated care with a biopsychosocial approach and strives to improve the Quadruple Aim outcomes: pain-related disability of patients with CMP; experiences of care of patients with CMP; meaning in the work of healthcare professionals; and healthcare costs. Firstly, in this study, the effectiveness (with regard to the functioning and participation of patients) of primary care for patients with CMP will be assessed, comparing care organized following the NPRL2.0 procedure with usual care. Secondly, the cost-effectiveness and cost-utility with regard to health-related quality of life and healthcare costs will be assessed. And thirdly, the effect of duration of participation in a local network in primary care will be studied.

**Methods:**

In this pragmatic study, it is expected that two local networks with 105 patients will participate in the prospective cohort study and six local networks with 184 patients in the stepped-wedge based design. Healthcare professionals in the local networks will recruit patients. Inclusion criteria: age ≥ 18 years; having CMP; willing to improve functioning despite pain; and adequate Dutch literacy. Exclusion criteria: pregnancy; and having a treatable medical or psychiatric disease. Patients will complete questionnaires at baseline (T1), 3 months (T2), 6 months (T3), and 9 months (T4). Questionnaires at T1 and T4 will include the Pain Disability Index and Short Form Health Survey. Questionnaires at T1, T2, T3, and T4 will include the EQ-5D-5L, and iMTA Medical Consumption and Productivity Cost Questionnaires. Outcomes will be compared using linear mixed-model analysis and costs will be compared using bootstrapping methods.

**Discussion:**

NPRL2.0 is a multidimensional, complex intervention, executed in daily practice, and therefore needing a pragmatic study design. The current study will assess NPRL2.0 with respect to the Quadruple Aim outcomes: patient health and costs. This will provide more information on the (cost-) effectiveness of the organization of care in a network structure regarding patients with CMP. The other two Quadruple Aim outcomes will be examined alongside this study.

*Trial registration* Netherlands Trial Register: NL7643. https://www.trialregister.nl/trial/7643.

## Background

In Western society, the prevalence of chronic musculoskeletal pain (CMP) is up to 20% in the adult population [1, 2]. CMP, the major cause of pain and disability, includes a diverse range of diagnoses such as nonspecific low back pain, fibromyalgia, complex regional pain syndrome, and nonspecific musculoskeletal pain [2, 3]. Biopsychosocial factors contribute to the development and persistence of pain and the associated perceived disabilities. However, the level of complexity of biomedical and psychosocial factors varies widely between people with CMP. This depends on the biomedical context and meaning of the pain, and on the impact of psychosocial factors, such as depression, anxiety, and social influences, on patients’ functioning [4, 5]. People with CMP often have difficulties in performing a range of daily activities and in maintaining an independent lifestyle. A high intensity of CMP is strongly associated with impaired function and is one of the leading causes of long-term work absenteeism and health-related early retirement, leading to high societal costs [6–10]. Earlier studies have shown that the health-related quality of life and levels of physical activity in people with CMP with a duration of 3–6 months is already low, and work absenteeism is high [1, 11, 12].

Due to high healthcare costs and high work absenteeism, CMP is one of the most expensive health conditions worldwide. In the Netherlands, CMP costs approximately 20 billion euros per year (direct and indirect costs) [11]. Of people with CMP, 60–74% receive treatment and most of these (34–79%) perceive the treatment as inadequate and therefore seek an explanation or solution for their pain problem [1, 13–15]. Earlier research shows that 61% of people with CMP had visited from six to more than 20 healthcare professionals in the year before starting a rehabilitation program [16]. A reason for medical ‘shopping around’ might be the more biomedical-oriented (instead of biopsychosocial-oriented) outlook of the general population, healthcare professionals, and decision-makers, in which explaining and solving the pain remains the ultimate focus [15, 17]. Additionally, healthcare professionals receive inadequate training on the assessment and management of CMP, leading to over- or under-treatment. As a result, the complexity of the patient’s pain problem does not accord with the treatment delivered [17–19]. This highlights the need for adequate (cost-) effective treatment strategies.

Multidisciplinary and interdisciplinary treatments, with a biopsychosocial focus in primary, secondary, and tertiary care, have been shown to be both clinically- and cost-effective for people with CMP [20–26]. In order to overcome the previously-mentioned challenges in rehabilitation care for people with CMP, a National Care Standard for Chronic Pain (NCSCP) was presented in the Netherlands in 2017 [11]. In this standard, a matched and person-centered care approach with multi- and interdisciplinary treatments in an integrated care network was proposed. This integrated care network would provide a shared vision of CMP and its biopsychosocial treatment through guidelines for referral and treatment. Moreover, there would be a focus on the early recognition of subacute pain in order to prevent this from becoming chronic. In line with this, the World Health Organization advises focusing on the stimulation of functioning and participation in the design of (new) rehabilitation care [27, 28].

As an elaboration of the NCSCP, the Network Pain Rehabilitation Limburg 1.0 (NPRL1.0) was developed to provide integrated care with a biopsychosocial approach for people with CMP in order to improve their level of functioning. Its main aim is to deliver the right care, at the right place, by the right person, for the right price, thus accomplishing the Quadruple Aim: improving the functioning and participation of people with CMP; improving the experiences of care of people with CMP; improving the meaning of the work of healthcare professionals; and reducing the healthcare costs of people with CMP [29, 30]. As a first step, a feasibility study was performed in 2017 and 2018 to assess the barriers and facilitators for the development, implementation, and transferability of NPRL1.0 [31]. The main facilitators were that the guidelines provide consistency and transparency in the collaboration of the healthcare professionals and that the iterative, bottom-up implementation strategy fits in with the target population with CMP. However, the current views and knowledge of CMP from the patient’s perspective, as well as from the healthcare perspective, and the current organization of care, are challenges for the implementation of NPRL1.0. The results of this feasibility study were used to adjust NPRL1.0 in areas such as the content of the education days for healthcare professionals, the eHealth application for healthcare professionals and patients, and educational information for patients, in the development of NPRL2.0 [32]. The existing local networks in primary care will participate in a cohort study in NPRL2.0. Additionally, extra local networks in primary care will be recruited. It is expected that healthcare professionals will experience a learning curve, as NPRL2.0 is a multidimensional, complex intervention [33]. Therefore, the long-term results of effectiveness, as well as views and knowledge, regarding CMP must be studied.

In this phase, the Quadruple Aim outcomes from NPRL2.0 will be evaluated. This study will focus on the (long-term) effectiveness, cost-effectiveness, and cost-utility part of the Quadruple Aim for primary care of patients with CMP organized according to NPRL2.0 compared to usual care. The research aims of this study are:

1. To evaluate whether primary care organized according to NPRL2.0 leads to a lower level of pain-related disability in patients with CMP than in patients receiving usual care (effectiveness).
2. To evaluate whether primary care organized according to NPRL2.0 is more cost-effective for the health-related quality of life in patients with CMP than in patients receiving usual care (cost-effectiveness).
3. To evaluate whether primary care organized according to NPRL2.0 leads to higher Quality Adjusted Life Years (QALYs) than in patients receiving usual care (cost-utility analysis).
4. To study the effect of duration of participation and the experience of using biopsychosocial principles in treatment of local networks on (cost)-effectiveness (learning curve).

## Methods

### Study design

In this pragmatic study, the recruiting period will be from April 2019 till March 2020, with follow-ups till December 2020. This study comprises two designs; a prospective cohort study and a stepped-wedge based design.

Two local networks of NPRL1.0 will be enrolled in NPRL2.0. They will receive additional education and information based on the results of the feasibility study of NPRL1.0. In NPRL2.0, they will invite patients to participate in a prospective cohort study.

**Fig. 1:**
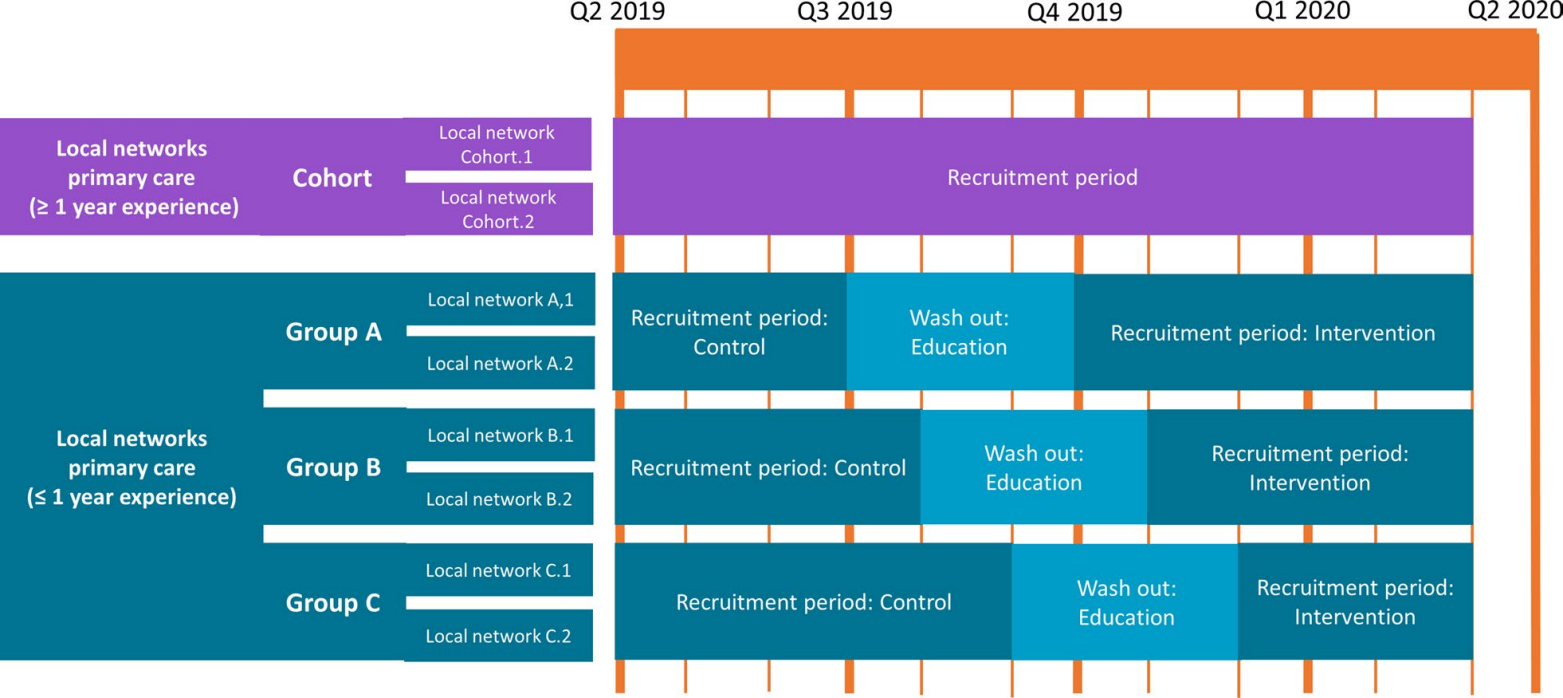
Design of the study. Cohort: prospective cohort; Group A, B, C: steps in the stepped-wedge based design. Q = quarter

In the stepped-wedge based design working according to NPRL2.0 will be introduced in three steps in two local primary care networks at the same step (step A, B or C). Local networks that intensively collaborate, due to their geographical location, will be placed together in one step (A, B or C). An independent research assistant will randomly allocate the local networks over the steps. In one local network, at least one therapist, general practitioner (GP), and mental health nurse will participate. Each local network will first recruit patients as controls during a period of care as usual, followed by a 3-month ‘wash-out’ period in which education is given (see Fig. 1). After the wash-out period, a local network will then recruit patients during the intervention period in which NPRL2.0 is the standard of care. According to the stepped-wedge based design, length of control and intervention periods vary in each group: Group A will spend 3 months as control and 5 months with intervention; for Group B, there will be 4 months as control and 4 months with intervention; and Group C will spend 5 months as control and 3 months with intervention. Thus, healthcare professionals in all local networks will recruit patients for participation in both control and intervention groups. Patients will contribute data to either the control group or the intervention group, but not both. A stepped-wedge based design is the most feasible design in this pragmatic study as it has the following advantages: (1) it controls for between-local network variation in daily practice; (2) it gives the opportunity to assess intervention effects in a pre/post comparison across local networks, which increases statistical power; (3) it gives an opportunity to assess learning effects by comparing the results of local networks that transit earlier with those that transit later [34].

### Intervention

**Fig. 2:**
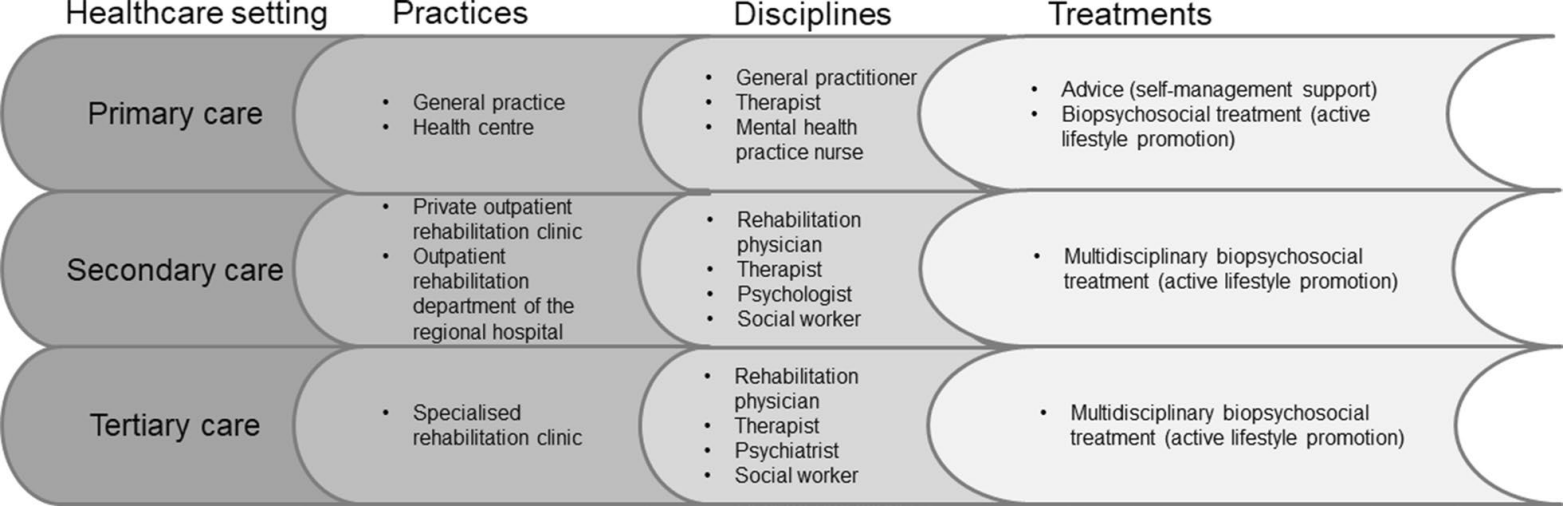
Referral options within Network Pain Rehabilitation Limburg: previously published in Lamper et al. [73]

NPRL2.0 is an integrated, transmural healthcare network for patients with CMP, focusing on improving the level of functioning of patients, despite pain. In the primary care of NPRL2.0, the GP is the gatekeeper for assessing the level of complexity of pain complaints, referral, and treatment selection. In the Netherlands, therapists (such as physiotherapists, practice therapists, and occupational therapists) in primary care can be visited by people with CMP directly, without referral. Therefore, therapists will also be able to assess the level of complexity of the pain complaints and to advise these patients to visit a GP if necessary. Depending on the level of complexity involved, the follow-up policy will either include advice without further treatment, monodisciplinary treatment in primary care, interdisciplinary treatment in primary care (collaboration between GPs, primary care therapists, and mental health practice nurses in assessing and treating patients with CMP who need mental support besides physical exercise) or interdisciplinary treatment in secondary or tertiary care (Fig. 2). Primary care in NPRL2.0 consists of the following elements:

#### Integral focus on assessment and referral: assessment tool

To support the healthcare professionals in their decision-making for problem-mapping and treatment selection, an evidence-based objective assessment tool will be used for the assessment of complexity of the pain problem: the STarT MSK Tool [35]. The Dutch version of this tool is translated and validated (not published yet). STarT MSK will support decision-making by choosing the right treatment to match the patient’s biopsychosocial profile.

#### Integral focus on treatment content and duration: treatment protocols

Patients will receive individualized treatments based on their current needs in order to improve their daily functioning. NPRL2.0 protocols are based on the most recent evidence-based treatment methods, such as Graded Activity, Exposure in vivo, and Acceptance and Commitment Therapy, and are adjusted to the primary care setting [22–24, 36, 37]. In the feasibility study, healthcare professionals were invited to provide feedback on the NPRL2.0 treatment protocols. Based on this, adjustments were made to the content and duration of NPRL2.0 treatment protocols. The primary care NPRL2.0 protocols are extended with a module focusing on self-management in daily living after treatment by a primary care therapist. In these treatment protocols, no advice for medication will be described. It is hypothesized that the biopsychosocial oriented healthcare professionals working in NPRL2.0 will prescribe less medication compared to patients receiving usual care.

#### Integral focus on self-management: eHealth application

Healthcare professionals and patients participating in the NPRL2.0 will make use of the eHealth application: SanaCoach Pain Rehabilitation [38]. The coach's primary goal is to support self-management. Its main function is to provide module-based pain education. Different eLearning modules are developed for the patients in order to teach them about the biopsychosocial aspects of pain. In addition, diaries are integrated in which patients can provide information on their pain intensity, level of activity, mood, and participation level. Moreover, healthcare professionals can use scores from these diaries to adjust treatment protocols to the needs of individual patients. The application also consists of a chat function between the patient and their healthcare professionals to ensure prompt communications. The functions in the SanaCoach Pain Rehabilitation, such as the number of diaries and the level of education, will be adapted to the patient, based on his/her complexity and level of disability.

#### Education and collaboration

Healthcare professionals will receive education during the 3-month wash-out period: GPs 2 × 3 h and therapists 3 × 3 h. Topics in the education program include biopsychosocial theories of CMP, recognition of patients with or at risk of CMP, providing education to patients with CMP, use of the assessment tool and eHealth application, and treatment selection. The first two sessions are organized jointly for all disciplines of healthcare professional in order to promote a common understanding of biopsychosocial treatment. Separately, therapists will also receive information about the treatment protocols. To encourage collaboration in the local networks, three additional peer-review meetings of one hour (every 6–8 weeks) are organized by the project team in each local network after the wash-out period. During these meetings, healthcare professionals apply the theories and treatment protocols learned during the education program in daily practice, with room for extra education by the teachers if necessary. After these three peer-review meetings, the local networks are encouraged to organize further such meetings in order to align the working procedures and treatment plans of the patients.

### Control

All networks start with a control period, in which local networks will invite patients who are attending consultations for CMP complaints to participate in the study. The healthcare professionals will refer and treat the patients, following the usual way of working in pain rehabilitation care in the Netherlands. In usual care, patients can receive treatments from a variety of approaches: from a more biomedical to a psychological or biopsychosocial approach. This results in a wide range of treatments that can vary in duration, content, and intensity, like medication prescription, a few sessions of physiotherapy in primary care or a complex multidisciplinary treatment in tertiary care. In usual care, the goal of the treatment does not have to be on daily functioning of the patient.

### Recruitment of primary healthcare professionals

Primary healthcare professionals (therapists, GPs and mental health nurses) working in the Parkstad region (Limburg, the Netherlands) who have no prior experience with NPRL1.0 will be recruited for participation in the study. Social media and the network of healthcare professionals of NPRL1.0 will be used to recruit new healthcare professionals. Healthcare professionals must be willing to recruit patients for the control and intervention periods, to attend the education days, and to make use of the assessment tool and treatment protocol of NPRL2.0.

### Recruitment of patients

Patients with CMP complaints, who visit the participating GPs and therapists via direct access, will be informed about the study and asked for consent to transfer their contact details to the research team. The research team will contact these patients by phone to inform them about the study and ask for oral consent for participation. Subsequently, the patients will receive the first questionnaire (T1) electronically or by post, in which they can give electronic/written informed consent for participation in the study.

Patients will be eligible if they are ≥ 18 years at the start of the study, have CMP or musculoskeletal pain with a high risk of becoming chronic, are willing to improve their functioning despite the pain, and have adequate Dutch literacy to complete the questionnaires. Exclusion criteria are pregnancy or any medical (orthopedic, rheumatic or neurological) or psychiatric disease which could be treated by a more appropriate therapy, according to the expert opinion of the GP. The data will be handled based on intention-to-treat.

### Sample size

In the prospective cohort, all patients with CMP who visit the two local networks of NPRL1.0 will be invited to participate in the study. Based on the recruitment results of the feasibility study of NPRL1.0, and the number of patients visiting a GP practice, we expect that each local network will also recruit about six patients per month. Therefore, the two local networks from NPRL1.0 together should recruit approximately 132 patients in 11 months. Assuming a dropout rate of 20%, we expect to include approximately 105 patients in this study.

To calculate the desired sample size for the stepped-wedge based design, we used the method described by Woertman et al. [39]. The calculations of the number of patients needed are based on the primary outcome of the cost-utility analysis, the health-related quality of life measured with the 5-level EQ-5D version (EQ-5D-5L). Based on McClure et al., we consider an increase of 0.063 points (SD = 0.013) in 1 year as clinically relevant [40]. In addition, an alpha of 0.05, a power of 80%, a 1:1 ratio between control and intervention groups, and a dropout rate of 30% were assumed. Based on these values and the stepped-wedge based design, a design effect (DEsw) of 0.416 exists, which leads to a required sample size of 184 patients (92 control and 92 intervention). Based on the recruitment results of the feasibility study of NPRL1.0, and the number of patients visiting a GP practice, we expect that each local network will recruit 6 patients per month [32]. Therefore, with a dropout rate of 30% of local networks, six local networks will need to participate.

### Data collection

**Fig. 3:**
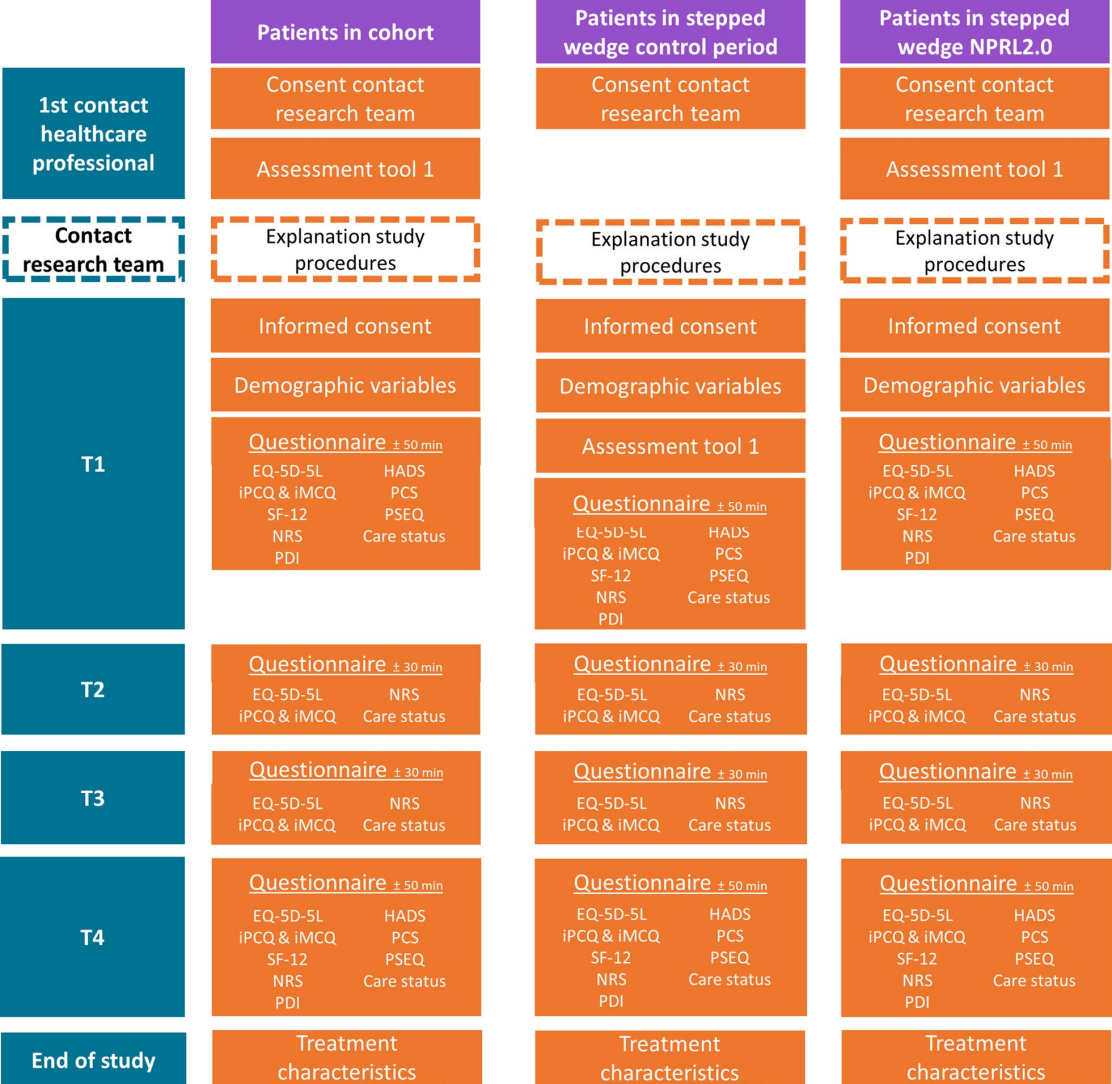
Content of data collection in ‘Patients in cohort’: patients participating in the prospective cohort design; ‘Patients in stepped-wedge based control period: data collection for patients participating in the control group of the stepped-wedge based design; ‘Patients in stepped-wedge NPRL2.0; data collection for patients participating in the intervention group of the stepped-wedge design

An overview of the content of the different data collection methods can be found in Fig. 3.

Patients participating in NPRL2.0 are asked to fill in four questionnaires electronically or on paper: T1 after initial contact with healthcare professionals about their CMP complaints (50 min completion time); T2 3 months after T1 (30 min completion time); T3 6 months after T1 (30 min completion time); and T4 9 months after T1 (50 min completion time).

Additionally, assessment tool 1 will be used for research purposes, as well as for decision-making in primary care. Therefore, patients in the control group will complete assessment tool 1 as part of the questionnaire at T1. Patients in the intervention group will complete it during their consultations in primary care. Assessment tool 1 will assess the level of complexity of the pain complaints and consists of one Visual Analogue Scale (VAS) for pain intensity and nine dichotomous questions on biopsychosocial factors.

At the end of the study, records of the treatment characteristics of each patient will be collected from the participating practices and rehabilitation centers.

In order to encourage completion of the questionnaires, patients will be reminded up to three times by phone if they have not responded within one week. If incomplete questionnaires are returned, patients will be contacted by phone to answer the remaining questions. The researcher who performs the analyses will be blinded as to patient allocation.

### Outcome measures

#### Baseline characteristics

Baseline characteristics will be collected at T1 and they will include questions about: birth date, gender, nationality, marital status, family composition, level of education, and co-morbidities.

#### Health assessment

The Pain Disability Index (PDI) will be used as the primary outcome for pain-related disability. It measures the influence of pain on a patient’s life and on the performance of daily activities. The questionnaire consists of seven items that measure the complexity of the disabilities experienced in different situations such as work, leisure time, activities in daily life, and sport. Each item is scored on a scale from 0 (no disability) to 10 (severe disability). Scores from the individual items are summed to a total (0–70). The minimal important change is 13 points for patients with CMP [41]. The Dutch version of the PDI has proven internal consistency and test–retest reliability [42].

The Dutch language version of the Short Form Health Survey (SF-12) will be used as the primary outcome for cost-effectiveness, measuring quality of life on specific domains. The SF-12 has proven to be a practical, reliable, and valid instrument for use in both general population surveys and in studies of chronic disease populations in the Netherlands [43, 44]. The SF-12 will be summarized into two scales: a physical component score (PCS) and a mental component score (MCS), in accordance with the guidelines for the SF12 instrument [45]. The PCS comprises the domains of physical functioning, physical role limitation, bodily pain, and general health perceptions. The MCS comprises the domains of vitality, social functioning, emotional role limitations, and general mental health. Both scores range from 0 to 100 (a higher score indicates a better quality of life) with a minimal clinically important difference of 8.9 for low back pain [46]. These sub-scales will be used in the effectiveness analysis. Besides the SF-12 score, the Short-Form Health Survey with six dimensions (SF-6D) scores will be used in a sensitivity analysis.

The EQ-5D-5L will be used for the cost-utility analysis: it provides a single health index based on self-reported mobility, self-care, usual activities, pain/discomfort, and anxiety/depression, with a minimal clinically important change of 0.04 [47]. There are five levels in each dimension from which respondents select that which most closely matches their health state. The levels are no, slight, moderate, severe, and extreme problems, coded 1 to 5. A health state index score, ranging from − 0.446 to 1 (worst to best imaginable health status), will be calculated from individual health profiles, using the Dutch utility tariff [48]. QALYs were calculated from utilities by using the area under the curve method. The accompanying visual analogue scale (VAS: 0–100) rates the current health state, with higher scores indicative of better experienced health. The minimal clinically important difference for low back pain is 22.5 [46]. The Dutch version of the EQ-5D-5L was found valid and reliable [49, 50].

The Numeric Rating Scale (NRS) will be used to measure pain intensity on an 11-point scale varying from 0 (no pain) to 10 (worst pain imaginable). At each measurement point (T1-4), the patient will complete the NRS three times: current pain intensity; lowest pain intensity in the last week; highest pain intensity in the last week. The NRS has shown high test–retest reliability and validity [51]. A reduction of 2 points, or 30%, on the pain NRS scores can be seen as clinically important [52].

The Hospital Anxiety and Depression Scale (HADS) consists of 14 items of which seven are related to anxiety and seven to depression. The patient is asked to rate the items on a 4-point scale ranging from 0 (not at all) to 3 (most of the time). Total scores range from 0 to 21 on each subscale: a higher score reflects higher distress. The HADS has a sensitivity and specificity of about 80% and a predictive validity for identification of about 70% [53]. The reliability ranges from 0.84 to 0.96 [54].

The six-item short form Pain Catastrophizing Scale (PCS-6) comprises six definitions of thoughts and feelings when experiencing pain [55]. The patient is asked to rate the definitions on a 5-point scale, ranging from 0 (not at all) to 4 (all the time), with total scores ranging from 0 to 24 [55]. The six-item version is used because it places a lower burden on patients than the original PCS. This form is adequate for detecting pre- to post-treatment changes in pain catastrophizing [56]. The PCS-6 is highly comparable to the original PCS and meets the construct validity criteria. Internal consistency and test–retest reliability of the original PCS appears to be adequate [55–57].

The Pain Self-Efficacy Questionnaire (PSEQ) is used to measure pain self-efficacy [58]. In patients with CMP, it shows satisfactory internal consistency and construct validity [59]. The four-item short form PSEQ-4 (items 4, 6, 8 and 9) will be used because it places a lower burden on patients than the original PSEQ [55]. Items are rated on a 7-point Likert scale ranging from 0 (not at all confident) to 6 (completely confident). The scores are summed, ranging from 0 to 24: the minimal important change is 1.5 points [60]. The PSEQ-4 is a good alternative for the PSEQ as the sensitivity and specificity of the PSEQ-4 are 0.803 and 0.687 respectively, compared with 0.648 and 0.875 respectively for the PSEQ [60].

#### Cost assessment

To evaluate the economic consequences of NPRL2.0 from a societal perspective, as recommended by the Dutch guidelines for costing studies in healthcare, the intervention costs, other healthcare costs, patient and family costs, and productivity losses will be assessed [48].

The intervention costs include costs of education meetings for healthcare professionals and peer review meetings for the intervention group, and consulting and/or treatment hours for the intervention and control groups. The education costs are for 2 × 3 h of education and 1 × 3 h of additional education for therapists. For each education session, the costs of two teachers and one meeting room will be taken into account. These costs will be charged at 10% per patient as it is assumed that healthcare professionals need education only once. Multidisciplinary consultations are organized with all healthcare professionals of the local networks in the absence of patients. For the multidisciplinary consultations per patient, the costs of the healthcare professionals will be divided by six, assuming that during one hour the status of six patients will be discussed. Moreover, it will be assumed that on average each patient is discussed during three multidisciplinary consultations. The number of consultations and/or treatment hours will be collected by the research team from the records of the patients in both the intervention and control groups. To calculate costs for healthcare professionals, standardized cost-prices as prescribed in the Dutch manual for cost-analysis in healthcare research will be used [48].

Healthcare usage will be measured with the iMTA Medical Consumption Questionnaire (iMCQ). It contains questions about healthcare consumption related to frequently-occurring contacts with healthcare professionals (www.imta.nl). The iMCQ will be combined with the iMTA Productivity Cost Questionnaire (iPCQ), a standardized instrument suitable for self-completion by patients for measuring and valuing all relevant productivity losses of paid and unpaid work for use in economic evaluations [61]. The manual for the iMCQ and iPCQ will be used for evaluating healthcare usage and productivity losses with the friction cost approach. The costs of prescribed medication will be calculated by multiplying the number of tablets that participants used during 3 months with the cost price as described at the Dutch webpage https://www.medicijnkosten.nl; the pharmacist costs will also be included. For over-the-counter medication, the lowest prices of Dutch drugstores and pharmacies will be used. All costs will be given in euros and, when necessary, indexed using the general Dutch Consumer Price Index rates [62].

Besides the iPCQ and iMCQ, the patients will be asked about their current care status and the treatment program for their CMP complaints. Moreover, at the end of the study, participating practices and rehabilitation centers will use the records of the patients to collect data about the length, content, and duration of the program.

#### Learning curve

Data regarding the background experience and knowledge of healthcare professionals will be assessed at the start of the study to judge whether there is a learning curve when participating in NPRL2.0. Whether patient outcomes regarding health and costs are improved when healthcare professionals have more experience of and knowledge about treating patients with CMP will be assessed.

The Pain Attitudes and Beliefs Scale (PABS) will be used to measure clinicians’ biomedical and biopsychosocial treatment orientations with respect to back pain [63]. It consists of 36 statements about treatment preferences, scored on a six-point Likert scale (from 1 = ‘totally disagree’ to 6 = ‘totally agree’). The sum score ranges from 6 to 60 for the biomedical factor and 6 to 54 for the biopsychosocial factor [64]. The PABS shows a consistent factor structure and good test–retest reliability and construct validity [65].

### Data analysis

Demographic data (e.g. gender, age, home situation, level of education, nationality, and co-morbidities) will be described overall and separately for the intervention and control groups. Frequencies are to be presented for categorical variables, means and standard deviations (SDs) for normally-distributed continuous variables, and medians and ranges for non-normally-distributed continuous data. The two groups will be tested on differences between characteristics, using the *t*-test for continuous variables and the chi-squared test for categorical variables. If variables differ between the two groups, with *p* ≤ 0.10, they are considered to be potential confounders in further analyses.

Outcomes on questionnaires will be compared using linear mixed-model analysis, to take into account repeated measurements in patients as well as the effects of the clustering of patients within local networks. The fixed part of the model contains treatment group (intervention/control), time, treatment group*time, and cluster (local network). To assess the learning effect in local practices, the time (months) that a local network participates in NPRL2.0 will also be taken into account as a fixed variable. Variables known to be related to the outcome and differing between treatment groups at T1 (*p* ≤ 0.10) will be added to the model. An unstructured covariance structure will be used for repeated measures. Missing values for items in the questionnaires will be handled according to the scoring algorithms of the questionnaires. Missing variables in the follow-up data will not be imputed because linear mixed-model analysis is a flexible method for handling missing data for stepped-wedge and repeated-measures designs (likelihood-based approach). Linear mixed-model analyses will be performed using IBM SPSS Statistics for Windows (version 24.0 or higher, Armonk, NY: IBM Corp.) according to the intention-to-treat principle. Other missing values for non-repeated measures will be handled by multiple imputation, which means that missing values will be predicted using existing values for other variables [66].

Costs will be compared using bootstraps (1000 replications) with Microsoft Excel 2016 with mean differences and 95% confidence intervals. Subsequently, sample uncertainties around the incremental cost-effectiveness ratio (ICER) and incremental cost-utility ratio (ICUR) will be explored using bootstrapping with a minimum of 5,000 replications. The ICER and ICUR will be defined by the difference in costs between NPRL2.0 and the control group, divided by the difference in incremental effects of the SF-12 and incremental QALYs respectively. Cost-effectiveness analyses will be performed with the mean total costs and the mean SF-12 scores. The cost-utility analysis will be performed by relating the mean total costs to the mean QALY scores of both groups, and the bootstrapped ICURs will be plotted in cost-effectiveness planes. Moreover, uncertainties of the ICERs and ICURs will be graphically presented in cost-effectiveness planes (CE plane), as well as cost-effectiveness acceptability curves (CEAC). A CEAC will be calculated to describe the probability of NPRL2.0 being a cost-effective alternative to the control group [67]. This CEAC includes the amount of money the society is willing to pay (WTP) in order to gain one unit of effect (one QALY here). The WTP threshold in the Netherlands for one QALY is based on the health burden and varies between €20,000 (health burden 0.1 to 0.4), €50,000 (health burden 0.41 to 0.7) and €80,000 (health burden 0.71 to 1) (2015) [68].

Four sets of sensitivity analyses will be performed to measure the robustness of the economic evaluation. These analyses will explore the impact of an assumption on the results when changing one value of one parameter while keeping all the other parameter values unchanged [69]. One sensitivity analysis will be performed to measure the influence of taking the educational costs included the intervention costs. Because healthcare professionals will only need training once, the intervention costs may be overestimated. The secondary sensitivity analysis will be performed to assess the influence of the multidisciplinary consultation costs. No standard cost price exists for multidisciplinary consultations in primary care in the Netherlands and it is not known how many patients will be discussed in order to be able to split the costs over these patients. The tertiary sensitivity analysis will be performed to see if there is over- or under-reporting of healthcare consumption in the iMCQ. The data from the records regarding GP and therapist sessions will be compared with the patient data from the iMCQ. When over- or under-reporting is found, a secondary cost analysis will be performed with corrections on all healthcare consumption data, assuming that the same amount of over- or under-reporting is present in the iMCQ. In a fourth sensitivity analysis the impact of the SF-6D to calculate QALYs instead of the EQ-5D-5L will be assessed.

## Discussion

The Network Pain Rehabilitation Limburg 2.0 (NPRL2.0) has been developed in order to provide integrated care with a biopsychosocial approach for people with CMP with the goal of improving their level of functioning. Moreover, it is intended to accomplish the Quadruple Aim: improvement of pain-related disability of people with CMP; improvement of experiences of care of people with CMP; improvement in the meaning of work for healthcare professionals; and the reduction of healthcare costs of people with CMP. In this quantitative study, the effectiveness of NPRL2.0 in reducing the pain-related disability of people with CMP will be assessed. In addition, the influence of NPRL2.0 on healthcare costs will be examined with a cost-effectiveness and cost-utility analysis. Moreover, the learning curve of healthcare professionals working in NPRL2.0 will also be studied.

NPRL2.0 is a multidimensional, complex intervention, executed in daily practice [33]. Because of the practice-based approach of this study, a randomized controlled trial design (RCT) is not suitable. Therefore, a pragmatic study with stepped-wedge based design using randomization of the local networks was seen as a viable alternative to an RCT [70]. The local primary care networks involved would be randomly assigned to the three steps (A, B or C) in order to randomize the duration of being a control group or intervention group. Local networks are their own controls in a stepped-wedge based design. Healthcare professionals are instructed to recruit patients at their first consultation for CMP complaints. Therefore, it is expected that patients with comparable complexities of complaints will be distributed equally over the control and intervention groups. Moreover, in this practice-based research, connections between science, policy, and practice exist during implementation and execution of NPRL2.0, leading to evidence-based practice. The external validity of the results of such as this pragmatic study of NPRL2.0 is commonly higher than that of RCTs because the results are more generalizable.

As NPRL2.0 is a complex intervention, it takes time for healthcare professionals to fully adopt the guidelines and treatments in their daily practice. Also, the internalization of the biopsychosocial perspective by healthcare professionals takes time and so no beneficial change in pain-related disability or healthcare costs is expected in the short term, as shown in other studies of complex interventions [71, 72]. Instead, the learning effect on the healthcare professionals will be studied. However, it is hypothesized that the effectiveness outcomes and healthcare costs, without the educational costs, will be no worse than with usual pain rehabilitation care. The results for the other Quadruple Aim outcomes, the experiences of care of people with CMP and meaning in the work of healthcare professionals, will be discussed elsewhere. These outcomes will be studied alongside this effectiveness and cost-utility study with a mixed-methods approach. A strength of this approach is that NPRL2.0 will be studied from different domains simultaneously.

## Data Availability

Not applicable.

## Abbreviations

CEAC: Cost-effectiveness acceptability curves
CE plane: Cost-effectiveness plane
CMP: Chronic musculoskeletal pain
DEsw: Design effect
EQ-5D-5L: 5-Level EQ-5D version
GP: General practitioner
HADS: Hospital Anxiety and Depression Scale
ICER: Incremental cost-effectiveness ratio
ICUR: Incremental cost-utility ratio
iMCQ: IMTA Medical Consumption Questionnaire
iPCQ: IMTA Productivity Cost Questionnaire
MCS: Mental Component Score
NCSCP: National Care Standard for Chronic Pain
NPRL: Network Pain Rehabilitation Limburg
NRS: Numeric Rating Scale
PCS-6: Pain Catastrophizing Scale 6-item
PDI: Pain Disability Index
PCS: Physical Component Score
PSEQ: Pain Self-Efficacy Questionnaire
QALY: Quality-Adjusted Life Year
RP: Rehabilitation physician
SD: Standard deviation
T1: First questionnaire
T2: Second questionnaire
T3: Third questionnaire
T4: Fourth questionnaire
SD: Standard deviation
SF-12: 12-Item Short Form Health Survey
SF-12 MCS: Mental health component scale
SF-12 PCS: Physical health component scale
WTP: Willing to pay
